# Supplementary material for: Ultrafast coupled charge and spin dynamics in strongly correlated NiO
Source: Nat Commun. 2020 Aug 14;11:4095. doi: 10.1038/s41467-020-17925-8 (PMC7429846; doi:10.1038/s41467-020-17925-8)
Supplement: Supplementary file 1 — Supplementary Information [file 41467_2020_17925_MOESM1_ESM.pdf]

Supplementary Information: Ultrafast coupled charge and spin dynamics in strongly correlated NiO

Konrad Gillmeister,[1] Denis Golež,[2,3] Cheng-Tien Chiang,[1] Nikolaj Bittner,[3]
Yaroslav Pavlyukh,[4] Jamal Berakdar,[5] Philipp Werner,[3] and Wolf Widdra[1,6]

[1]*Institute of Physics, Martin-Luther-Universität Halle-Wittenberg, 06120 Halle, Germany*

[2]*Center for Computational Quantum Physics, Flatiron Institute, 162 Fifth Avenue, New York, NY 10010, USA*

[3]*Department of Physics, University of Fribourg, 1700 Fribourg, Switzerland*

[4]*Department of Physics, Technische Universität Kaiserslautern, 67653 Kaiserslautern, Germany*

[5]*Institute of Physics, Martin-Luther Universität Halle-Wittenberg, 06120 Halle, Germany*

[6]*Max Planck Institute for Microstructure Physics, 06120 Halle, Germany*

(Dated: July 15, 2020)

I. SUPPLEMENTARY NOTE 1: EXPERIMENTAL DETAILS

a. Structural, magnetic and electronic characterization The NiO(001) thin films have been prepared by reactive metal deposition in an oxygen atmosphere. The films have been characterized by low-energy electron diffraction (LEED) for a sharp NiO(001)-(1×1) pattern. The NiO thin film growth on Ag(001) has been studied complementarily by scanning tunneling microscopy (STM), scanning tunneling spectroscopy (STS), and high-resolution electron energy loss spectroscopy (HREELS)[1–4]. The inset in Supplementary Figure **1** shows an atomically resolved STM image of a 4 ML NiO film on Ag(001), which resolves the (1×1) structure. The differential conductivity $\mathrm{d}I/\mathrm{d}V$ measured under constant tunneling current conditions is depicted in Supplementary Figure **1** for a 6 ML NiO(001) film. It shows pronounced density-of-states features below and above the Fermi energy that can be directly compared with photoemission and 2PPE spectra. The STS peak marked V corresponds to the screened $3d^8\underline{L}$ final state as discussed in the context of Fig. 1 in the main text. The two peaks above the Fermi energy marked S and V correspond to electron tunneling into the NiO $3d_{z^2}$ surface state predicted by theory[5,6] and into the UHB. Note that due to dielectric screening within the NiO film, we expect that the STS peak energies for unoccupied (occupied) states are slightly up (down) shifted as compared to field-free conditions in photoemission.

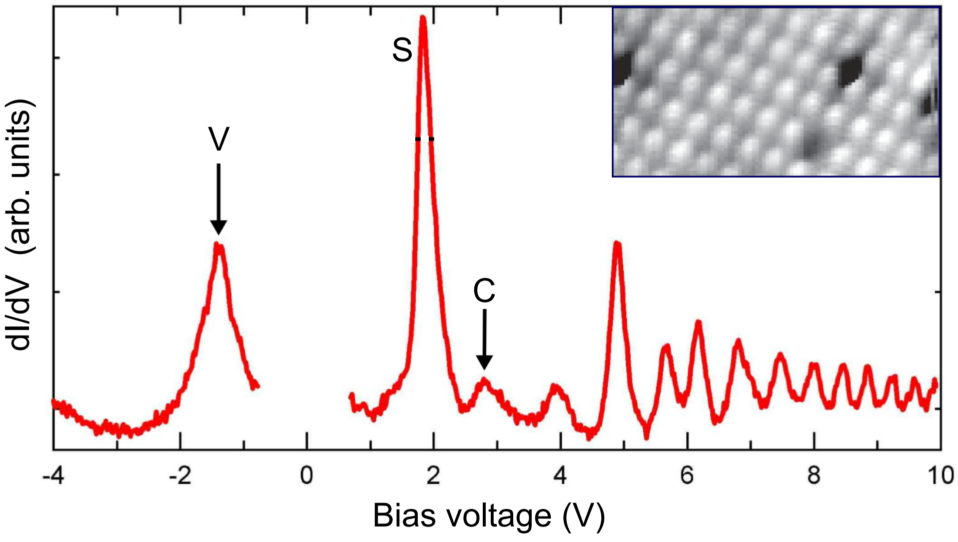

Supplementary Figure 1. Differential conductivity as determined by STS under constant current condition for 6 ML NiO(001) on Ag(001) (80 K). **Inset**, Atomically resolved STM image for 4 ML NiO(001) on Ag(001) (1.8 x 3.5 nm, 80 K).

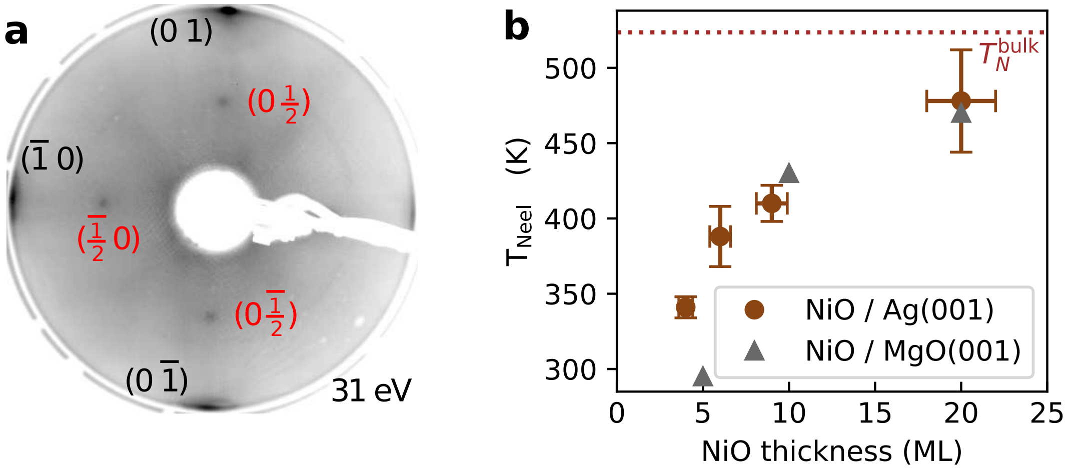

Supplementary Figure 2. **a**, LEED pattern of a 4 ML NiO(001) thin film on Ag(001) for an electron kinetic energy of 31 eV. At this energy, the half-order spots due to the magnetic (2×1) superstructure (marked in red) are clearly visible. Note that the first order diffraction spots are located at the rim of the LEED screen. **b**, Extracted Néel temperatures versus NiO film thickness (brown circles). Literature data from Ref. 7 for NiO films on MgO(001) are marked by grey triangles.

The STS features above 4 eV are dominated by field emission resonances.

To assure a long-range antiferromagnetic order, the weak (2x1) superstructure due to the doubled antiferromagnetic unit cell[8] has been analyzed as depicted for a 4 ML film at room temperature in Supplementary Figure **2**(a). At a kinetic energy of 31 eV, the weak but sharp half-order spots are clearly discernible. The integrated half-order spot intensity is strongly temperature dependent and vanishes at the Néel temperature as discussed in the main text for NiO(001) films with 4, 9, and 20 ML thickness. The Néel temperature as determined from the vanishing magnetic (2x1) signal is shown in Supplementary Figure **2**(b) as brown circles for all investigated film thicknesses. In addition, data for NiO films on MgO(001) from Ref. 7 are marked by grey triangles. In agreement with earlier studies, we find that the Néel temperature is reduced for ultrathin films as compared to $T_N = 523.6$ K for NiO bulk[9–11]. The relative temperature error is about 5 K; the absolute error is estimated to be 25 K. The (2x1) LEED spots are generally weak which introduces errors for the LEED intensities as given by the scatter of the data.

A detailed comparison of the temperature dependence of the THz oscillation lifetime and the magnetic scatting amplitude as derived from the (2x1) LEED spots is presented in the main text and yields critical temperatures T' of 473 and 565 K for 9 and 20 ML thick films, respectively. There we demonstrated a $(1 - T/T')^{0.64}$ temperature dependence as experimentally found earlier

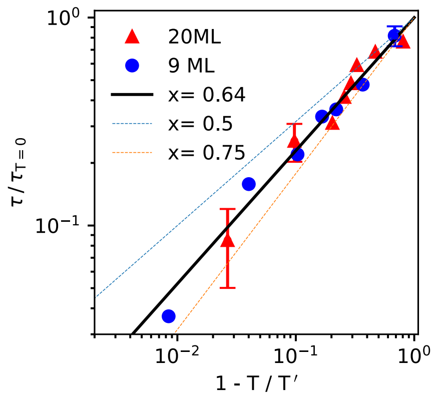

Supplementary Figure 3. Double logarithmic plot of the relative THz oscillation lifetime for 9 and 20 ML thick NiO(001) films on Ag(001) as function of $(1 - T/T')$. The straight lines indicate $(1 - T/T')^x$ power laws for $x = 0.5$, 0.64, and 0.75. The best fit to the data corresponds to $x = 0.64$, in agreement with neutron data[11].

for the short-range spin correlation length based on neutron scattering[11]. The critical exponent in our experiment is analyzed in Supplementary Figure **3** by the double logarithmic plot of the THz oscillation lifetime versus $1 - T/T'$. The best fit yields an exponent of 0.64 ± 0.07 as indicated by the solid line.

b. Time-resolved two-photon photoemission (2PPE) The time-dependent 2PPE signal at the bottom of the UHB (2.5 eV above E_F) is depicted in Supplementary Figure **4**(a) for a 9 ML thick NiO(001) film on a logarithmic intensity scale. Here pump and probe photon energies of 4.11 and 3.31 eV, respectively, have been used. The time-dependent signal follows directly the experimental cross correlation trace, which is shown as dashed curve and has a full-width half maximum (FWHM) of 69 fs. We estimate from this observation that the signal decays within a time shorter than 10 fs. Similar fast decays have been found for all investigated film thicknesses as summarized in Supplementary Figure **4**(b).

The 2PPE spectrum for the 10 ML NiO film is depicted in Supplementary Figure **5** (right axis) as a gray trace for a monochromatic excitation with $h\nu_{\text{pump}} = h\nu_{\text{probe}} = 3.88$ eV. As discussed in the main manuscript, excitations across the charge-transfer gap lead to long-lived in-gap states that are located about 1 eV below the UHB and exhibit characteristic oscillations in their photoemission intensities. The amplitude of these oscillations is shown in Supplementary Figure **5** (red and black

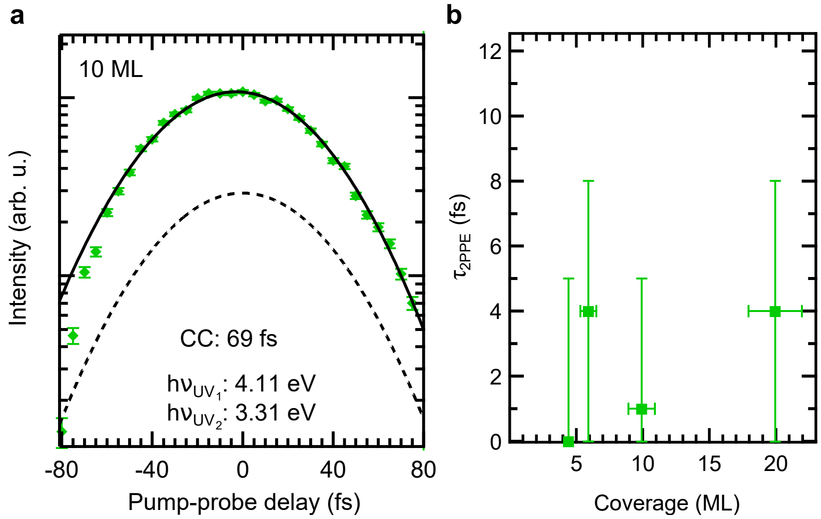

Supplementary Figure 4. **a**, Time-dependent 2PPE intensity 2.5 eV above E_F (bottom of the upper Hubbard band) as a function of the pump-probe delay for a 10 ML thick NiO film on Ag(001). The cross correlation of the pump and probe pulses (dashed line) has been determined from the 2PPE signals at higher energies. **b**, Extracted lifetime of the 2PPE signal at the bottom of the UHB for NiO thin films between 4 and 20 ML thickness. In all cases, the lifetime is below 10 fs.

traces, left axis) as a function of the intermediate state energy for four different photon energies. The 2PPE oscillation amplitude is determined around a pump-probe delay of Δt= 248 fs, which corresponds to the first oscillation maximum. Supplementary Figure **5** shows that in all cases the oscillations are observable in the intermediate state energy range between 1 and 1.5 eV above E_F, independent of the final state energy. The oscillation amplitude as function of the laser fluence is depicted in Supplementary Figure **6** for a 9 ML NiO(001) thin film. For a fixed pump fluence, the amplitude scales proportionally with the probe fluence (red squares). For a fixed probe fluence, it scales proportionally with the pump fluence as marked by the blue circles.

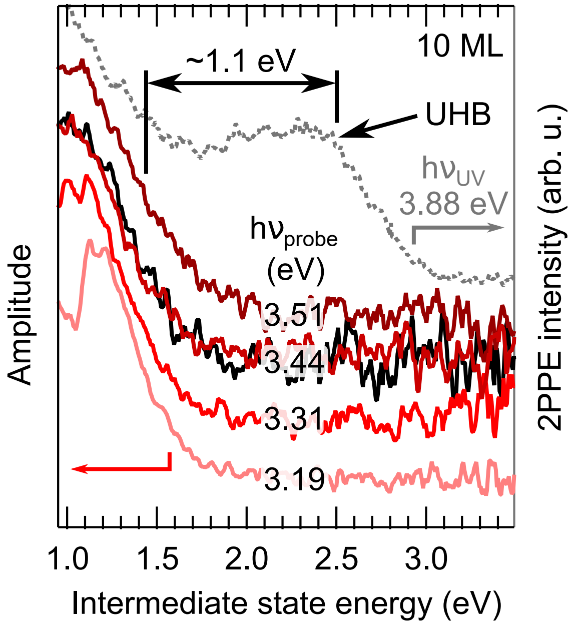

Supplementary Figure 5. Amplitude of the 2PPE oscillations as a function of the intermediate state energy after pumping with $h\nu_{\mathrm{pump}} = 4.11\,\mathrm{eV}$ (light red curves) and $h\nu_{\mathrm{pump}} = 3.95\,\mathrm{eV}$ (black curve). The probe photon energy $h\nu_{\mathrm{probe}}$ is varied between 3.19 and 3.51 eV as indicated in the graph. The grey photoemission spectrum is obtained by monochromatic 2PPE ($h\nu_{\mathrm{pump}} = h\nu_{\mathrm{probe}} = 3.88\,\mathrm{eV}$) and is provided to indicate the position of the UHB. Note that the THz oscillations start about 1 eV below the bottom of the UHB. The spectra are shifted vertically for clarity.

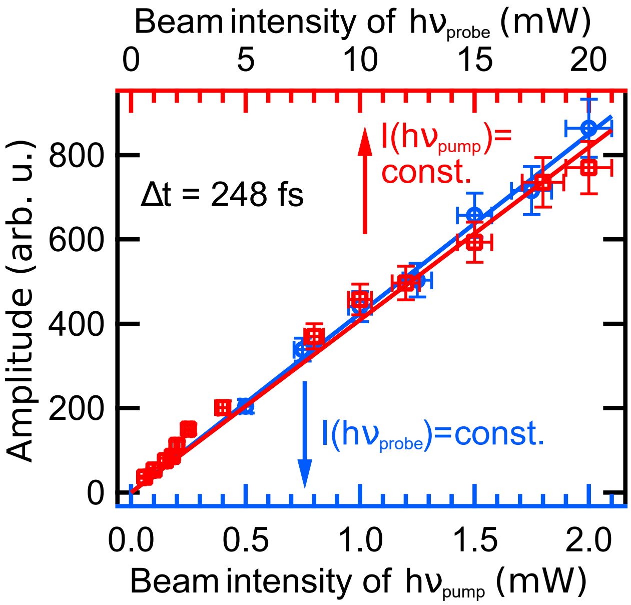

Supplementary Figure 6. Amplitude of 2PPE oscillations as a function of the laser pump ($h\nu_{\mathrm{pump}} = 4.17\,\mathrm{eV}$) and probe ($h\nu_{\mathrm{probe}} = 3.39\,\mathrm{eV}$) intensities for a 9 ML thick NiO film on Ag(001). The time delay between the pump and probe beams is fixed to 248 fs. The oscillation amplitude scales linearly with both intensities.

II. SUPPLEMENTARY NOTE 2: NONEQUILIBRIUM SPECTRAL FUNCTION AND RELAXATION MECHANISMS

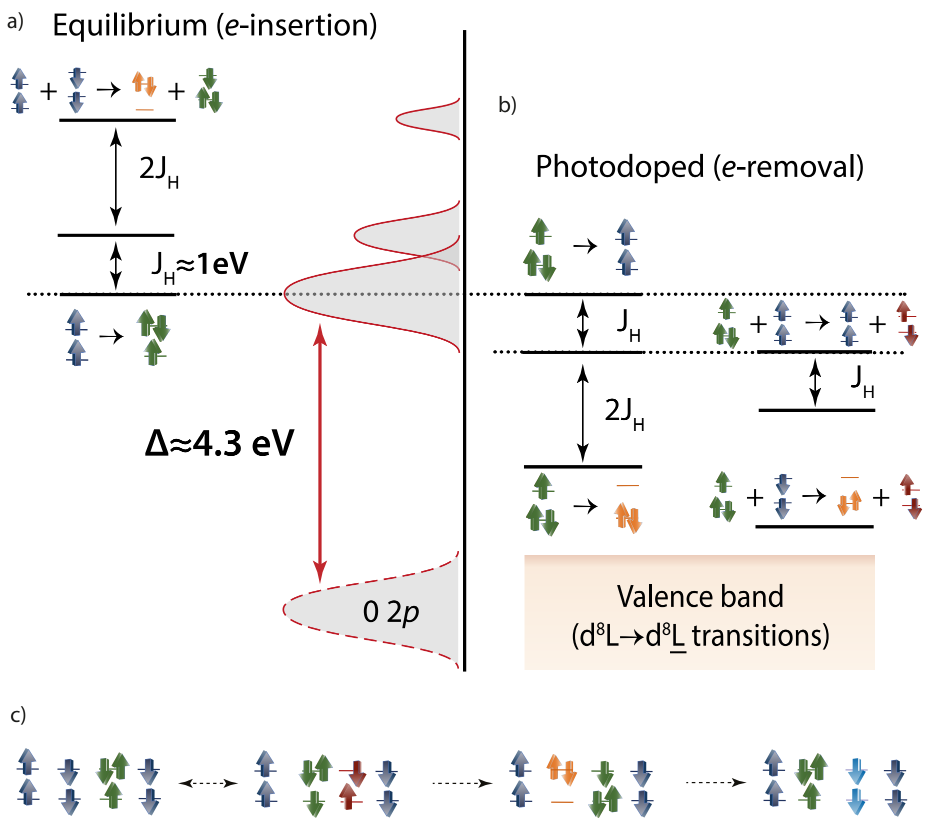

Supplementary Figure 7. **a**, Electron transitions in the inverse photoemission from the ground state. **b**, Electron transitions in the transient photoemission from a photodoped state. Excitations on the neighboring site lead to a shifted ladder of states. **c**, Schematic view of the decay channel for coherent oscillations. Transient coherent oscillations can decay via the creation of a high-energy Hund excitation $S0^\flat$ and the formation of a ferromagnetic domain wall.

The time-dependent spectral function $A(\omega, t)$ and the photo-emission spectrum $\mathrm{PES}(\omega, t)$ are the main observables that we use for the interpretation of the time-resolved two-photon photoemission (tr-2PPE). In equilibrium and for periodic driving the spectral function can be expressed in the Fermi Golden rule form allowing for the scattering theory interpretation. In this interpretation, each spectral peak is associated with a particular scattering process (electronic transition from the ground N-particle state to excited $N \pm 1$ particle states). This approach can be further extended to the transient regime by including the excited-excited transitions. Our goal here is to provide

additional information on the transient $A(\omega, t)$ and PES(ω, t) depicted in Fig. 4 of the main text from this perspective.

In Supplementary Figure **7**(a,b) we depict scattering mechanisms pertinent to the equilibrium and excited (photodoped) states of the system. In the panel (a) we focus on the inverse photoemission part of the spectrum. The upper Hubbard band (UHB) is predominantly formed by the $d^8 \to d^9$ transitions visible as strong broad peaks (4.5 to 6 eV) in the equilibrium $A(\omega, t)$ (main text, Fig. 4(a), black line). For instance, the dominant subband near 4.5 eV can be associated with triplon formation on a S1 site, while the spectral feature with energy $\approx 3J_H$ above this UHB edge results from an electron insertion followed by a hopping process which leaves behind a S0$^\flat$ state. These electron insertion and Hund excitation processes are accompanied by the electron hopping processes in the antiferromagnetic background — the t-J model physics. Some of the resulting side-bands can be identified as weak shoulders below 4 eV in Fig. 4(a).

The lower Hubbard band (LHB) has a complicated structure even for the ground state. This has been the subject of numerous theoretical (Bała, Oleś and Zaanen[12]) and experimental (Taguchi *et al.*[13]) investigations. The current state-of-the-art knowledge is that LHB is formed by the $d^8 \to d^7$ local transitions, the charge transfer transitions $d^8L \to d^8\underline{L}$, and the Zhang-Rice bound state $3d^8\underline{Z}$. Since we do not focus explicitly on this part of the spectrum (discussed in details in the main part, Fig.1), we denote all these features as a shaded area in Supplementary Figure **7**(b). The *photo-induced in-gap states*, resulting from the electron removal from a triplon state (T) and visible as featureless spectral density in the range of 2 to 4 eV and two broad peaks at 0.5 and 1.5 eV, are of the prime interest to us because they serve as intermediate states for tr-2PPE (panel b).

Let us thus focus on the PES signal in Fig. 4(b), where these states are well-visible. The conceptual difference with the spectral function [Fig. 4(a)] is that the former measures only the populated states as encoded in the lesser Green's function ($G^<$), whereas the latter describes all spectral density (populated and un-populated) as encoded in the retarded Green's function (G^R). The photo-induced in-gap states visible in PES can be explained as follows:

1. There are three spectral lines originating from the local transitions: T$\to$ S1, S0, S0$^\flat$.

2. Local transitions may be accompanied by excitations on the neighboring sites. They lead to a *shifted ladder* of spectral lines, as depicted in the right column of Supplementary Figure **7**(b).

3. Triplons may decay over a variety of intermediate states by eventually dispersing all the

energy to magnetic degrees of freedom, i.e., emitting string excitations, Supplementary Figure **7**(c).

The six spectral lines from 1 and 2 merge into five, as is indeed seen in Fig. 4(b). But what about the fine structure on the exchange energy scale? The respective hopping processes are also depicted in the right column of Supplementary Figure **7**(b). These satellites are impossible to discern from the main lines in the full energy- and time-resolved PES. Therefore, we integrate over an energy window (black bar in Fig. 4(b)) and plot the Fourier transform of the time-signal in Fig. 4(d). Here, different peaks appear, which can be classified according to the number of Hund and string excitations in the initial and final state, respectively. The former scale with J_H and represent very fast oscillations which cannot be experimentally resolved. The latter ones are observed as oscillations in the tr-2PPE signal and are the main subject of this study.

---

[1] S. Großer, C. Hagendorf, H. Neddermeyer, and W. Widdra, Surf. Interface Anal. **40**, 1741 (2008).

[2] K. L. Kostov, S. Polzin, S. K. Saha, O. Brovko, V. Stepanyuk, and W. Widdra, Phys. Rev. B **87**, 235416 (2013).

[3] K. Kostov, F. Schumann, S. Polzin, D. Sander, and W. Widdra, Phys. Rev. B **94**, 075438 (2016).

[4] K. Gillmeister, M. Kiel, and W. Widdra, Phys. Rev. B **97**, 085424 (2018).

[5] D. Ködderitzsch, W. Hergert, W. M. Temmerman, Z. Szotek, A. Ernst, and H. Winter, Phys. Rev. B **66**, 064434 (2002).

[6] A. Schrön, M. Granovskij, and F. Bechstedt, J. Phys. Condens. Matter **25**, 094006 (2013).

[7] D. Alders, L. Tjeng, F. Voogt, T. Hibma, G. Sawatzky, C. Chen, J. Vogel, M. Sacchi, and S. Iacobucci, Phys. Rev. B **57**, 11623 (1998).

[8] P. Palmberg, R. DeWames, and L. Vredevoe, **21**, 682 (1968).

[9] M. Marynowski, W. Franzen, M. El-Batanouny, and V. Staemmler, Phys. Rev. B **60**, 6053 (1999).

[10] S. Altieri, M. Finazzi, H. H. Hsieh, M. W. Haverkort, H.-J. Lin, C. T. Chen, S. Frabboni, G. C. Gazzadi, A. Rota, S. Valeri, and L. H. Tjeng, Phys. Rev. B **79**, 174431 (2009).

[11] T. Chatterji, G. J. McIntyre, and P.-A. Lindgard, Phys. Rev. B **79**, 172403 (2009).

[12] J. Bała, A. M. Oleś, and J. Zaanen, Phys. Rev. B **61**, 13573 (2000).

[13] M. Taguchi, M. Matsunami, Y. Ishida, R. Eguchi, A. Chainani, Y. Takata, M. Yabashi, K. Tamasaku, Y. Nishino, T. Ishikawa, Y. Senba, H. Ohashi, and S. Shin, Phys. Rev. Lett. **100**, 206401 (2008).
